# Pain Management in Knee Osteoarthritis: Insights from an Exploratory Online Survey of Italian Patients and Physicians

**DOI:** 10.3390/healthcare12202077

**Published:** 2024-10-18

**Authors:** Giovanni Iolascon, Alberto Migliore, Giovanna Beretta, Andrea Bernetti, Roberto Bortolotti, Antonella Celano, Antonino Giarratano, Franco Marinangeli, Alberto Momoli, Gian Domenico Sebastiani, Andrea Tomasini, Silvia Tonolo, Fabiana Madotto, Alberto Di Martino, Silvia Natoli

**Affiliations:** 1 Department of Medical and Surgical Specialties and Dentistry, University of Campania “Luigi Vanvitelli”, 80138 Naples, Italy; 2 Rheumatology Unit, San Pietro Fatebenefratelli Hospital, 00189 Rome, Italy; 3 Unit of Rehabilitation Medicine and Neurorehabilitation, Department of Neuroscience, ASST Niguarda Hospital, 20162 Milan, Italy; 4 Department of Experimental Medicine, University of Salento, 73100 Lecce, Italy; 5 Unit of Rheumatology, Santa Chiara Regional Hospital, Azienda Provinciale Servizi Sanitari, 38122 Trento, Italy; 6 Italian National Association People with Rheumatological and Rare Diseases, APMARR Aps, 73100 Lecce, Italy; 7 Department of Precision Medicine in Medical, Surgical and Critical Care (Me.Pre.C.C.), University of Palermo, 90127 Palermo, Italy; 8 Department of Anesthesia, Intensive Care and Emergency, Policlinico Paolo Giaccone, 90127 Palermo, Italy; 9 Department of Anesthesiology, Intensive Care and Pain Treatment, University of L’Aquila, 67100 L’Aquila, Italy; 10 Unit of Trauma and Orthopaedic, San Bortolo Hospital, 36100 Vicenza, Italy; 11 Rheumatology Unit, Azienda Ospedaliera San Camillo-Forlanini, 00152 Rome, Italy; 12 ANMAR Onlus (Associazione Nazionale Malati Reumatici), 00145 Rome, Italy; 13 Department of Anesthesiology, Intensive Care and Emergency, Fondazione IRCCS Ca’ Granda Ospedale Maggiore Policlinico, 20122 Milan, Italy; 14 First Orthopaedic Department, IRCCS-Istituto Ortopedico Rizzoli, 40136 Bologna, Italy; 15 Department of Biomedical and Neuromotor Sciences—DIBINEM, University of Bologna, 40126 Bologna, Italy; 16 Department of Clinical-Surgical Diagnostic and Pediatric Sciences, University of Pavia, 27100 Pavia, Italy; 17 Unit of Pain Therapy Service, Foundation Istituto di Ricovero e Cura a Carattere Scientifico (IRCCS), Policlinico San Matteo, 27100 Pavia, Italy

**Keywords:** knee, osteoarthritis, pain, patient perspective, qualitative research, osteoarthritis management

## Abstract

Background: In Italy, knee osteoarthritis (KOA) accounts for over 5 million prevalent cases and requires long-term multidisciplinary management. The most frequent KOA symptom is pain. The aim of the survey is to provide a national overview of the current management of moderate-to-severe pain associated with KOA from both clinicians’ and patients’ perspectives. Methods: An exploratory qualitative survey was conducted in Italy between July and October 2023. The survey participants were members of four national scientific societies and two patient associations who were invited to participate via email and given an online questionnaire. Questions included a mix of single and multiple responses and scalar items. Results: In total, 1473 clinicians and 150 patients with KOA completed the questionnaire. Patients rated knee pain as both the most burdensome symptom and the most frequent reason for treatment dissatisfaction and seeking consultation. One in two patients declared that they were unsatisfied/little satisfied with the current prescribed analgesic treatments. The clinicians surveyed preferentially prescribed intra-articular hyaluronic acid, oral non-steroidal anti-inflammatory drugs/paracetamol, non-pharmacological intervention, and intra-articular corticosteroids. The clinicians’ selection of analgesic therapy often failed to adequately meet patients’ expectations for pain relief. Conclusions: Our findings highlight the current unmet needs of patients with KOA in Italy and call for new approaches to effectively optimize the management of KOA-associated pain.

## 1. Introduction

The knee is the most common site of osteoarthritis (OA); indeed, knee OA (KOA) accounts for almost four fifths of the total OA burden [1]. KOA is highly prevalent, mostly in the female sex, and is one of the largest contributors to combined OA age-standardized prevalence at the global level [2]. Current estimates predict that approximately 642 million individuals will have a diagnosis of KOA by 2050 [2]. Thus, KOA stands as an alarming global health priority because of the associated functional disability and chronic pain and the socioeconomic implications, including absenteeism, early retirement, impaired productivity, and quality-adjusted life year loss [3,4]. The most frequent symptom of KOA is knee pain, for which the presentation and characteristics vary widely, ranging from constant to intermittent, sharp or dull, and from mild to severe. Knee pain is one of the main contributors to the reduced range of motion observed among patients with KOA and, ultimately, to the functional limitations in daily living activities and impaired quality of life [5]. Among patients with KOA, knee pain is the main reason for seeking medical advice [6]. The Framingham Osteoarthritis Study reported that age and body mass index-adjusted knee pain prevalence was greater than 25% (32.9% in women and 27.7% in men) [7].

KOA is a progressive multifactorial joint disease, and it is very often accompanied by comorbid chronic diseases; as a result, patients with KOA more frequently present with other musculoskeletal conditions, including rheumatic diseases, followed by type 2 diabetes, hypothyroidism, peripheral vascular disease, and other comorbidities [3]. KOA treatment should be instituted as early as possible to relieve pain and inflammation, reduce stiffness, and improve or preserve the range of motion [4,8,9]. Although KOA treatment choice should consider disease characteristics, patients’ expectations and preferences, and clinician recommendations [10], one in two European patients with KOA suffer from inadequate pain relief, display moderate-to-severe pain [11], and are unsatisfied with the prescribed pain-relieving interventions [12]. In line with this, a recent survey conducted among patients with OA, 60% of whom had KOA, reported that patients were poorly satisfied with their current treatment plan while seeking access to additional drug treatments. These results suggest the urgent need for a new integrated approach and novel medications to effectively target KOA [12].

In Italy, the KOA burden significantly affects the national healthcare system and society. KOA accounts for over 5 million prevalent cases and an age-standardized prevalence rate of 4066 cases per 100,000 [2]. As an additional marker of KOA burden in our country, almost 139,000 individuals (65% women) underwent total knee arthroplasty between 2020 and 2021 [13]. Throughout the disease, KOA can be characterized by different degrees of severity, thus demanding long-term multidisciplinary management with the involvement of different clinicians. In our country, the first-line approach to KOA has mostly been managed by general practitioners (GPs). In contrast, the second and third-level approach involves specialists, such as pain therapists, rheumatologists, orthopedics, and physiatrists [14,15]. However, except for local experiences, shared clinical pathways in KOA management are lacking in Italy. Notably, although national scientific societies have developed consensus and position statements for non-surgical KOA management [15,16,17], different treatment strategies are currently adopted in routine practice for the same pathology grade. As a result, patients with KOA bounce from one specialist to another until they obtain the analgesic treatment deemed appropriate for them. Therefore, gaining insights from the various specialists caring for patients with KOA can shed light on both potential gaps in KOA care in Italy and strategies to effectively control KOA-associated pain. As recommended by national and international guidelines [4,15,16,17], initial KOA management should be conservative, requiring both a pharmacological and a non-pharmacological approach. Given the wide variety of available treatments, several national scientific societies developed a conjoined effort to provide practical guidance to prescribing physicians [15,16,17]. However, data on how this evidence-based knowledge has been implemented in real life are scant in Italy. Moreover, the perspective of Italian patients affected from KOA requires further investigation. Overall, a national snapshot of treatment patterns and patient care pathways in KOA management is urgently needed. To this end, a joint initiative involving four Italy-based scientific societies and two patient associations was established in 2023 to gather insights into the national scenario via an online survey. The survey aimed to explore both clinicians’ and patients’ perspectives on the current KOA management, focusing on managing moderate-to-severe pain associated with the disease.

## 2. Methods

### 2.1. Survey Design

The survey was carried out across Italy from 5 July to 15 October 2023. The survey involved members from the national scientific societies actively engaged in the management of KOA, namely the Italian Society of Anesthesia, Analgesia, Resuscitation, and Intensive Care (SIAARTI), the Italian Society of Rheumatology (SIR), the Italian Society of Orthopedics and Traumatology (SIOT) and the Italian Society of Physical and Rehabilitative Medicine (SIMFER). The scientific societies have been recognized by the *Istituto Superiore di Sanità* (Italian National Institute of Health, the main center for research, control, and technical-scientific advice on public health in Italy) according to the Law 24/2017 and DM 02.08.2017 [18]. Furthermore, to explore patients’ perspectives on KOA management, two patient associations, namely the Italian Association of People with Rheumatological and Rare Diseases (APMARR) and the Italian Association of Rheumatological Patients (ANMAR), were engaged to contribute to the survey. The sample size was calculated by considering the following factors: the number of specialists in each category reachable through the Italian scientific societies involved in the survey, the desired precision in the final estimates set at 5%, and the anticipated dropout rate (individuals not completing the questionnaire) set at 10% for all the professionals included. A sampling convenience strategy was adopted as previously described [19]. Given finite survey populations, a sample size for each medical profession and patient association was estimated to be 356, 354, 407, 393, and 424 for SIAARTI, SIR, SIOT, SIMFER, and patients suffering from KOA (APMARR and ANMAR), respectively.

### 2.2. Survey Development

A steering committee encompassing eight clinicians identified as experts in the field by the respective scientific societies (SIAARTI, SIR, SIMFER, SIOT) and two patient representatives of the patient associations (APMARR, ANMAR) gathered on 14 June 2023 to identify the survey items and draft both the clinician and patient questionnaires. A biostatistician reviewed the questionnaires to assess the content, clarity, and readability of each survey item. The questionnaires were created with an exploratory intent, specifically for the Italian reality. Questions included a mix of single and multiple responses and scalar items. The final version of the survey questionnaires was shared with the steering committee before the online administration for final approval.

The clinician questionnaire comprised 41 multiple-choice questions addressing the following items: (a) participant demographics; (b) clinical profile and therapies taken by the patients encountered by participating clinicians in their daily practice; (c) prescribing habits of clinicians when dealing with patients with KOA; (d) degree of satisfaction/dissatisfaction with the therapeutic approaches employed to relieve the moderate-to-severe pain associated with KOA. The patient questionnaire comprised 31 multiple-choice questions addressing the following items: (a) participant demographics; (b) patient clinical profile and symptom impact on daily life; (c) patient perspective on their disease course: time to diagnosis, relationship with clinicians, treatment patterns, degree of satisfaction/dissatisfaction with prescribed therapy. Participants were asked to rate how much symptoms like pain would impact their daily life by using a 0–10 point Likert scale (0 = no impact; 10 = burdensome). Participants were asked to indicate the intensity of their pain by using a 0–10 point Likert scale (0 = no pain; 10 = worst pain). Participants were asked to rate their satisfaction/dissatisfaction from very unsatisfied to very satisfied (0, very unsatisfied; 1, unsatisfied; 2, neutral; 3, satisfied; 4, very satisfied) on a 5-point Likert-type scale.

Hereby, we report an aggregate analysis of the answers of clinicians to whom the online questionnaire was administered regardless of their specialty. Of note, the reported findings from both clinician and patient questionnaires stem from 30 out of 41 (clinician questionnaire) and 23 out of 31 (patient questionnaire) questions, which are reported in the Supplementary Materials.

### 2.3. Data Collection

Clinicians affiliated with SIAARTI, SIOT, SIR, and SIMFER and patients belonging to APMARR and ANMAR were invited to participate via email and given an online questionnaire. No inclusion/exclusion criteria were established. The online questionnaire was administered through a computer-aided web interview using the SurveyMonkey Enterprise platform (Momentive, San Mateo, CA, USA). Answers were collected anonymously. All respondents provided voluntary, informed consent to data collection and use based on a clear understanding of the purpose of the survey. Data were collected from an online platform complying with applicable privacy regulations, such as the General Data Protection Regulation (GDPR).

### 2.4. Data Analysis

Data were downloaded as an Excel file (Microsoft Corp., Redmond, WA, USA) and analyzed using the SAS software (version 9.4) and R software (version 4.3.1) for descriptive statistics. Answers were included in the analysis if participants responded to questions from the demographic section and at least one question from the other questionnaire sections. Missing answers were included in the analysis. The exclusion criteria included duplicated answers from the same participant. Data are presented descriptively as numbers, mean ± standard deviation (SD), and median (range).

## 3. Results

A total of 1473 clinicians and 150 patients with KOA completed the questionnaire. Table 1 and Table 2 illustrate the demographics of the patient and clinician respondents, respectively. The patients surveyed (81% were women) had a median age of 57 years, and one in two presented with OA at both knees. Beyond knees, patients reported being affected from OA at other joints, mostly the hip, which is followed by the hand/wrist, lumbar, cervical, shoulder, ankle, and elbow. One in two patients (49.3%) declared a disease duration longer than 5 years and was diagnosed with KOA at a median age of 49 years. Hypertension was found to be the most frequent comorbidity among the patients surveyed, second only to a variety of rheumatological conditions, including arthritis, spondylarthritis, thyroid disease, and fibromyalgia. Patients received their first diagnosis of KOA mostly by orthopedics and rheumatologists and, on average, encountered at least three clinicians before the first diagnosis. More than half waited for less than 1 month for the first therapy, while one in four (26%) had to wait for more than 6 months to be prescribed the first therapy (Table 1).

The clinicians surveyed (62% were men) had a median age of 45 years and were mostly distributed in Northern Italy (49%). Of the 1473 physicians completing the questionnaire, 1147 were specialists and 326 were residents. Among specialists, almost one in two (47.8%) reported ≥20 years of medical practice. The clinicians surveyed were members of SIAARTI (n = 438), SIOT (n = 612), SIMFER (n = 311), and SIR (n = 112). The clinicians surveyed reported working in different types of healthcare facilities, the most common being universities and public hospitals. On average, most clinicians (62.5%) saw 25–100 patients with OA monthly, and among them, the occurrence of KOA (primarily II–III Kellgren–Lawrence grade and mostly caused by primary degenerative OA) was the most frequent (31%) (Table 2). Of note, a large proportion of the patients encountered by the clinicians surveyed had already received a KOA diagnosis from orthopedics, which was followed by GPs (Table 2). Clinicians who treated an undiagnosed patient reported that the time from diagnosis to prescription of the first therapy was no longer than 1 month in more than 80% of cases. This contrasts with the observation that up to 45% of patients received their first therapy more than 1 month after the diagnosis (Table 1).

**Figure 1:**
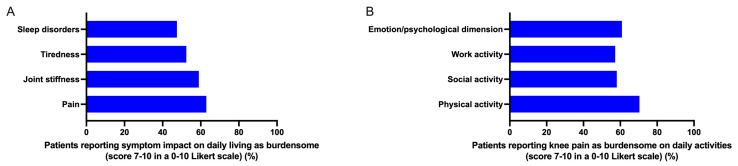
Impact of KOA symptoms (panel **A**) and associated pain (panel **B**) on patients’ daily life.

**Figure 2:**
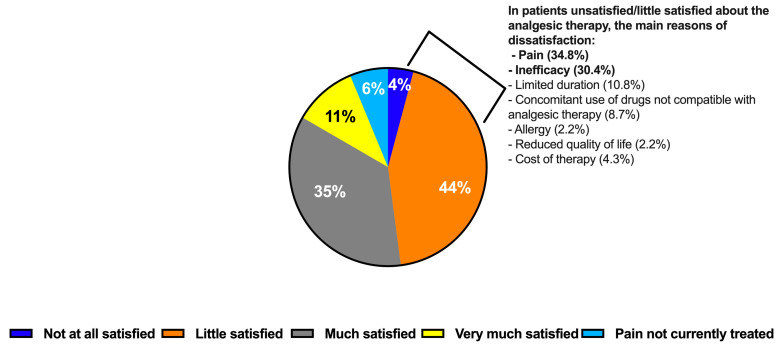
Patients’ degree of satisfaction with analgesic therapy and reasons of dissatisfaction as reported by the patients surveyed.

Patients reported that the most burdensome (score 7–10 on a 0–10 Likert scale) symptoms were pain and joint stiffness, which were followed by tiredness and sleep disorders (Figure 1A). The patients deemed these symptoms to have the most severe impact on their daily lives. Knee pain was found to significantly impair physical activity, work, and social activity as well as have a major impact on patients’ emotions and psychological well-being (Figure 1B). Overall, 75% of the patients surveyed rated their pain intensity as moderate to severe (score ≥ 4 on a 0–10 Likert scale) with one in two patients reported suffering from severe pain (score 7–10 on a 0–10 Likert scale). About two thirds (60%) of the patients the clinicians surveyed encountered in their daily practice presented with pain of moderate-to-severe intensity, which was a lower proportion than that of the patients surveyed who had a long-term rheumatological condition, severe OA-associated pain, and on chronic OA therapy. The patients surveyed declared that pain was the primary reason for seeking medical advice. They reported that the most frequent analgesic interventions were oral non-steroidal anti-inflammatory drugs (NSAIDs), non-pharmacological therapies (e.g., physical therapy, lifestyle changes), and intra-articular hyaluronic acid (IAHA) (Table 1). However, one in two patients declared that they were unsatisfied/little satisfied with the current prescribed treatments (Figure 2). Pain and inefficacy were listed as the most frequent reasons for the dissatisfaction of patients with KOA with the prescribed analgesic therapy (Figure 2). Of note, pain was listed as the most frequent reason for patients with KOA to consult the clinicians surveyed (Table 2).

**Figure 3:**
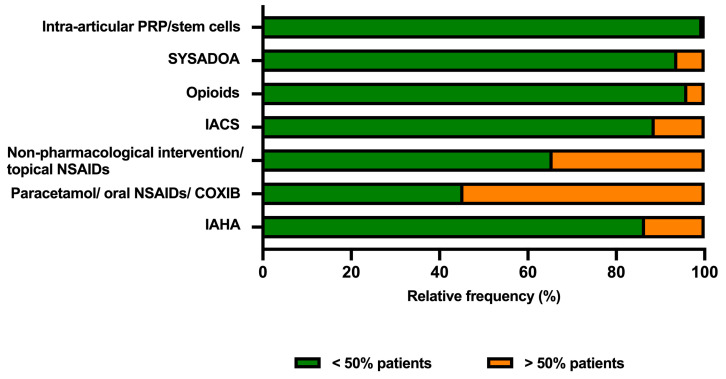
The most frequent analgesic interventions prescribed to patients with KOA with moderate-to-severe pain as reported by the clinicians surveyed who encountered patients for referral. COXIB, cyclo-oxygenase inhibitor; IACS, intra-articular corticosteroids; IAHA, intra-articular hyaluronic acid; NSAIDs, non-steroidal anti-inflammatory drugs; PRP, platelet-rich plasma; SYSADOA, symptomatic slow-acting drugs for osteoarthritis.

In daily practice, the clinicians surveyed would encounter patients with a prior KOA diagnosis and on pain therapy as well as patients for whom they should prescribe a pain-relieving drug. As shown in Figure 3, the clinicians surveyed reported that the most frequent (in more than 50% of patients) analgesic interventions they prescribed to patients with KOA with moderate-to-severe pain were paracetamol/oral NSAIDs/cyclo-oxygenase inhibitor (COXIB) (54.6%), non-pharmacological intervention/topical NSAIDs (34.4%), IAHA (13.5%) and intra-articular corticosteroids (IACS) (11.3%).

**Figure 4:**
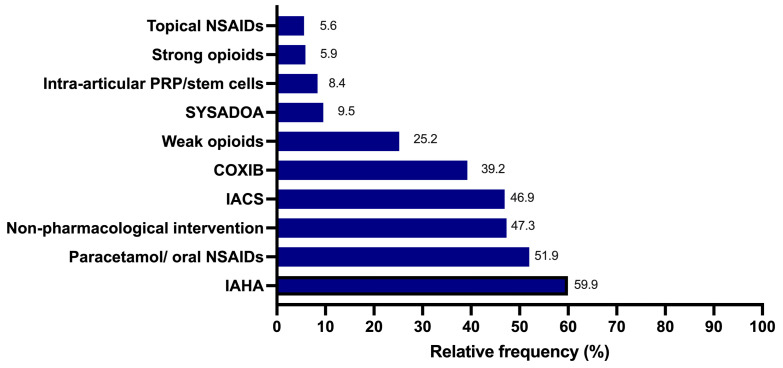
Most frequent prescribed analgesic interventions as reported by the clinicians surveyed when managing a patient with moderate-to-severe KOA-associated pain. COXIB, cyclo-oxygenase inhibitor; IACS, intra-articular corticosteroids; IAHA, intra-articular hyaluronic acid; NSAIDs, non-steroidal anti-inflammatory drugs; PRP, platelet-rich plasma; SYSADOA, symptomatic slow-acting drugs for osteoarthritis.

To explore the prescribing patterns of the clinicians surveyed when treating KOA-associated pain, they were asked to list the three most frequent interventions of their choice. As shown in Figure 4, IAHA, oral NSAIDs/paracetamol, non-pharmacological intervention, and IACS were the first-line approaches chosen by clinicians, which was followed by COXIB, weak opioids, and symptomatic slow-acting drugs for osteoarthritis (SYSADOA). Interestingly, the clinicians surveyed preferred intra-articular injections and non-pharmacological therapies more than the physicians who cared for the patients before referral.

**Figure 5:**
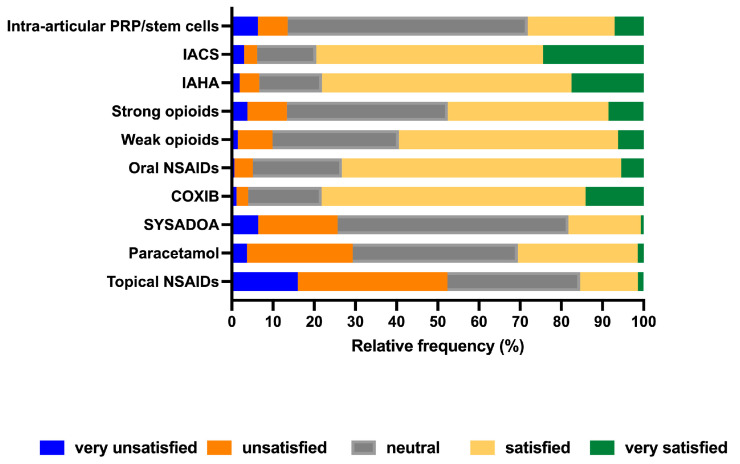
Clinicians’ degree of satisfaction with the pain relief obtained with currently available analgesic interventions. COXIB, cyclo-oxygenase inhibitor; IACS, intra-articular corticosteroids; IAHA, intra-articular hyaluronic acid; NSAIDs, non-steroidal anti-inflammatory drugs; PRP, platelet-rich plasma; SYSADOA, symptomatic slow-acting drugs for osteoarthritis.

Clinicians were also asked to rate their satisfaction with the pain relief obtained from the treatments they regularly prescribed to patients with KOA. As shown in Figure 5, the greatest degrees of satisfaction (very satisfied/satisfied) were observed with IACS (79%), which was followed by COXIB (78%), IAHA (78%), and oral NSAIDs (73%). More than half of clinicians reported being very satisfied/satisfied with weak (59%) and strong opioids (47%). However, such classes of medications were less prescribed than intra-articular injections or oral NSAIDs/COXIB. The observed pattern of satisfaction among clinicians contrasts with the lower degree of satisfaction reported by the patients surveyed (Figure 2).

To investigate further the experience of care provision for patients with KOA, we delved into the communication between patients and clinicians. Upon diagnosis, 43% of patients felt that clinicians insufficiently informed them about the disease, lifestyle advice, strategies for managing the disease self-efficiently, and the available therapeutic options, thus feeling left out of the decision-making process. Accordingly, almost half of patients (47.6%) felt their expectations for pain relief were not satisfactorily addressed by the clinicians’ therapy choice. In contrast, most clinicians reported that they valued patients’ expectations much/very much when choosing analgesic therapy during the pain management associated with KOA. Finally, patients listed the following as the most relevant gaps in their diagnostic-therapeutic pathways: (a) access to treatment along with the need to wait for a long time to receive a diagnosis with the likelihood of encountering more than one clinician to obtain a diagnosis (20.8%); (b) uncertain diagnoses and disagreement in therapy selection among the clinicians encountered (16.2%); (c) inadequate pain control and challenges in identifying effective pain-relieving approaches (11.7%).

## 4. Discussion

With over 14 million individuals over 65 years as of 2023 and the highest median age (48.4 years) in the European Union, Italy will need to face the burden of aging-associated conditions, including KOA, in the coming years [20,21]. This paper provides the first overview of the current management of pain associated with KOA in Italy from both patients’ and clinicians’ perspectives. Unsatisfactory pain control is common among patients with KOA, and our patient population displays many predictors of inadequate pain relief, including female sex, obesity, longer OA duration, and bilateral KOA [11]. The observation that 75% of patients rated pain intensity as moderate to severe suggests that pain treatments currently prescribed for KOA may not be deemed to adequately meet the needs of most patients with moderate-to-severe pain associated with KOA. Mounting evidence from clinical trials supports the efficacy of therapies usually suggested as first- and second-line treatments for KOA [22,23,24,25,26]. However, the unsatisfactory pain relief reported by the patients surveyed seems to suggest that the effectiveness of available analgesic therapies may vary over time and eventually decline. In our clinical experience, it is not unusual that despite the validity of analgesic therapies, the efficacy of prescribed medications may not be long-lasting; thus, due to the worsening of osteoarticular degenerative pathology, analgesic drugs and infiltrations may lose effectiveness in advanced stages of the disease.

In fact, not all analgesic medications are regarded as equally effective and suitable for long-term therapy. For instance, in patients with KOA, NSAID-induced improvement in pain and function peaks at 2 weeks and wanes over time by 8 weeks [27]; therefore, patients reporting oral NSAIDs as the most frequently prescribed medication may have experienced short-term pain relief that later led to patient dissatisfaction with the prescribed therapy. Our data showed that NSAIDs are widely prescribed to the patient population surveyed; however, only 11% of the clinicians surveyed reported inflammation as the reason patients encountered in their clinical practice would seek consultation. Therefore, we can speculate that in our patient population, the use of NSAIDs might be less preferable than other treatment options. This observation is relevant when considering that the appropriate use of this class of analgesic medications is not always made in OA management. In our country, the initial therapy with NSAIDs is more frequently switched to weak opioids than other initial drug selections, thus suggesting that NSAIDs are also commonly prescribed for moderate-to-severe pain despite their limited efficacy [28]. Although clinicians prescribing NSAIDs are aware of the importance of reducing or preventing their untoward effects [29], they are quite satisfied with the pain relief obtained with such medications (73%). Of note, NSAIDs are still perceived as easier to manage than opioids, as dose titration and potential tapering are not required. Nevertheless, oral NSAIDs and opioids were found equally effective in reducing KOA-associated pain with no difference in the Western Ontario and McMaster Osteoarthritis Index pain subscale [30]. Interestingly, national recommendations regarding the use of opioids are not consistent among specialists; indeed, orthopedics recommend their use only for short term and while waiting for arthroplasty [16], while rheumatologists consider them when pain is severe and NSAIDs are not tolerated or are contraindicated [17]. In Italy, opioids are poorly prescribed as the first therapy in patients with KOA; however, when patients experience severe pain despite long-term therapy, opioids are more often chosen by the clinicians surveyed who, in great proportion, declared being very satisfied/satisfied with the opioid-induced pain relief. The prescribing pattern stemming from the responses of the clinicians surveyed clearly highlights the frequent use of intra-articular injections, mostly HA and CS-based, to control moderate-to-severe pain, despite the potential risk of joint deterioration and worsening symptoms over the long term recently observed following the use of IACS [31,32,33]. The observed prescribing pattern differs from the latest OA Research Society International (OARSI) guidelines, which conditionally recommended IACS for acute and short-term pain relief and IAHA for longer-term treatment effect, as it was associated with symptom improvement beyond 12 weeks and demonstrated a favorable safety profile [4]. Of note, a position paper from a multidisciplinary panel of orthopedics, rheumatologists, and physiatrists underlined that IAHA can be repeated safely, while they advised caution in considering the repeated use of IACS [15]. Nevertheless, as recalled by the SIR guidelines, the accuracy of intra-articular injection depends on the joint and on the skills of the practitioner [17]. Symptom control can be slow acting and achieved with SYSADOA use, which is supported by positive experiences in clinical practice [9] and indicated as a step 1 approach by the European Society for Clinical and Economic Aspects of Osteoporosis, Osteoarthritis and Musculoskeletal Diseases (ESCEO) guidelines [8]. In our study, SYSADOAs were prescribed less frequently for the management of KOA-associated pain than other recommended treatment options [proportion of clinicians reporting use in more than 50% of patients was 6.2% vs. 54.6% (oral NSAIDs/paracetamol/COXIB) and vs. 13.5% (IAHA)]. This finding could be related to the contrasting recommendations by ESCEO vs. the European Alliance of Associations for Rheumatology (EULAR) [34,35]; thus, making the use of SYSADOA in KOA still controversial. Finally, although recommended as a first-line approach in national [15,16,17] and international guidelines [4,8], non-pharmacological intervention was less frequently indicated in the patient history than the recommended second-line approach, such as NSAIDs or paracetamol.

Evidence-based clinical practice guidelines are an effective tool to improve patient care quality. The latest OARSI and EULAR guidelines encourage clinicians to continually provide patients with necessary information about OA disease progression and self-care techniques [4,36]. However, one in two patients surveyed felt that clinicians did not sufficiently inform them regarding the disease, lifestyle advice, and strategies to manage the disease self-efficiently. In line with previous qualitative studies [37,38], patients with KOA felt that their complaints were not taken seriously, and their expectations were not effectively addressed by the prescribed medications. Furthermore, we report differences between patients’ expectations for information and engagement in the clinical decision-making process and clinicians’ perceptions of their commitment to ensuring patients’ needs are fully met by prescribed interventions. Overall, the views of the patients with KOA and clinicians participating in the survey differ greatly regarding their perception of disease burden and their assessment of what is important in health and symptom status. To further explore the magnitude of perception gaps between patients with KOA and clinicians who care for them, it would be relevant to design studies enrolling GPs, as they are the potential first clinicians to care for patients with KOA in the general population. Additional potential insights may come from surveys involving patients with hypertension and/or renal impairment, for whom analgesic medication selection should consider potential contraindications. The evidence stemming from these studies may help reconcile the contrasting views and foster interventions to better implement patients’ perspectives in KOA care pathways. Finally, our findings highlight a high degree of dissatisfaction of patients with KOA with the prescribed pain-relieving therapy with pain being the most relevant contributor followed by inefficacy, therapy duration, cost of therapy and concomitant treatments. We acknowledge that patients’ dissatisfaction regarding the prescribed pain medications can be influenced by a wide range of confounding factors including female gender, socioeconomic status, and significant comorbidities. Nevertheless, our findings unveil the need to pursue a more person-centered approach. To this end, studies including patient-reported outcomes developed for the relevant assessment of pain in KOA, such as the Patient Acceptable Symptom State, along with other well-established patient-reported outcome measures, i.e., the Western Ontario and McMaster Osteoarthritis Index, the Short Form 36 and the Knee Disability and Osteoarthritis Outcome Score, are urgently awaited. To date, the Patient Acceptable Symptom State allows patients to be classified as either in “an acceptable state” or not, thus placing the patient at the center of clinical decisions in managing osteoarticular conditions [39]. Overall, such studies can better inform clinical decisions when patients report inadequate pain control despite the long-term use of analgesic medications.

### Limitations

The patient sample comprised members of APMARR and ANMAR who are rheumatological patients with multi-joint OA and comorbidities. Therefore, the patients surveyed may be less representative of patients with KOA within the general population. The finding that participants surveyed, being mainly rheumatological patients, displayed a rheumatological condition as a second comorbidity may be biased by their membership to specific patient associations, namely APMARR and ANMAR. In addition, the patients surveyed were not managed by the clinicians surveyed and differed from those they encountered daily. However, given the chronic nature of KOA symptoms and the resulting need for long-term therapeutic solutions, we felt it was highly relevant to explore the view of patients undergoing long-term treatment for other diseases. We selected this patient population in line with the relevance of stratifying treatment recommendations for co-morbidities and the presence of OA in joints other than the knee, as advocated by the OARSI KOA type classification [40]. Of note, the degree of dissatisfaction with the prescribed therapy reported by patients receiving long-term analgesic treatment reinforces the need to place more emphasis on seeking novel pain-relieving approaches that can ensure long-lasting pain control while ameliorating functional disability associated with KOA. In addition, as patterns of care and treatment options differ across countries, our findings stemming from a convenient national sample of clinicians and patients may not be fully generalizable to other national healthcare systems. To this end, conducting studies in other geographical areas with similar approaches would be useful to compare results and broaden the generalizability of our findings. Also, online surveys can be subjected to response and self-selection bias, and online questionnaires can be associated with gender and age-related biases. Of note, the cross-sectional design of the study does not allow us to effectively capture the longitudinal effects of KOA patients’ care. Finally, for some medical professions, the minimum sample size was not reached [namely, SIR (112 vs. 354) and SIMFER (311 vs. 393)], and this may imply a lower accuracy of the results presented. Nevertheless, our findings contribute to fostering awareness of the burden of KOA in our country and unveiling the current unmet needs of patients with KOA.

## 5. Conclusions

The findings of our study provide relevant insights into the needs and priorities of patients with KOA as well as barriers to care within our national healthcare system to better shape KOA management pathways from the patient’s perspective. Our findings call for new approaches to effectively optimize the management of KOA-associated pain. Collectively, our results laid the foundation for further investigations to promote patient–clinician relationships, identifying the weaknesses of the current standard of care and optimizing the diagnostic and therapeutic journey for patients with KOA.

## Figures and Tables

**Table 1:** Demographics of survey participants (Patients).

Respondents (n = 150)	
**Gender, n (%)**	
**M**	26 (17.33)
**F**	122 (81.33)
**Not reported**	2 (1.33)
*Geographical origin, n (%)*	
*Northern Italy*	63 (42)
*Central Italy*	33 (22)
*Southern Italy*	54 (36)
**Age (years)**	
*Mean ±* SD	54.54 ± 10.86
Median [IQR]	57 [47–62]
Minimum–Maximum	25–77
**Age groups, n (%)**	
≤55 years	75 (46.01)
≥55 years	88 (53.99)
**Membership to patient organizations, n (%)**	
ANMAR	45 (30)
APMARR	26 (17.33)
Not reported	79 (52.67)
**Knee OA, n (%)**	
Both knees	66 (55.46)
One knee	53 (44.54)
**Joints affected from OA beyond the knee, n (%)**	
Hip	49 (32.67)
Lumbar	41 (27.33)
Cervical	36 (24)
Shoulder	33 (22)
Elbow	17 (11.33)
Hand/wrist	42 (28)
Ankle	27 (18)
None	59 (39.33)
**Disease duration, n (%)**	
<6 months	12 (8)
6–12 months	12 (8)
1–3 years	30 (20)
3–5 years	22 (14.67)
>5 years	74 (49.33)
**Age at knee OA diagnosis**	
*Mean ±* SD	*46.26 ±* 12.69
Median [IQR]	49 [40–54]
Minimum-Maximum	11–72
**Comorbidity, n (%)** ^a^	
Hypertension	35 (29.41)
Diabetes	7 (5.88)
Cardiovascular disease	12 (10.08)
Obesity	25 (21.01)
Other *	70 (58.82)
None	26 (21.85)
**Clinician making knee OA diagnosis, n (%) ^a^**	
Orthopedics	54 (45.38)
Rheumatologist	46 (38.66)
Physiatrist	9 (7.56)
Radiologist	7 (5.88)
General practitioner	2 (1.68)
Pain therapist	1 (0.84)
**Time from diagnosis to first therapy, n (%) ^a^**	
Immediately	36 (30.25)
<1 month	29 (24.37)
1–3 months	15 (12.61)
3–6 months	8 (6.72)
>6 months	31 (26.05)
**Knee OA diagnosis made by means, n (%) ^a^**	
Magnetic resonance	62 (52.10)
X-rays	41 (34.45)
Physician objective examination	7 (5.88)
Echography	3 (2.52)
Not remembered	6 (5.04)
**Current analgesic intervention for knee OA, n (%) ^§^**	
Non-pharmacological therapies	41 (42.71)
Topical NSAIDs	11 (11.46)
Oral NSAIDs	48 (50)
Paracetamol	13 (13.54)
Intra-articular hyaluronan	20 (20.83)
Intra-articular corticosteroids	6 (6.25)
Other #	5 (5.21)
None	6 (6.25)

* Other included: arthritis (32.77%), spondylarthritis (6.72%), thyroid disease (10.92%), and fibromyalgia (5.88%). # Other included biologics, immunosuppressors, cortisone, supplements, and surgical intervention. ^§^ the respondents were 96. ^a^ the respondents were 119.

**Table 2:** Demographics of survey participants (clinicians).

Respondents (n = 1473)	
**Gender, n (%)**	
**M**	918 (62.32)
**F**	550 (37.34)
**Not reported**	5 (0.34)
**Geographical origin, n (%)**	
*Northern Italy*	721 (48.95)
*Central Italy*	357 (24.24)
*Southern Italy*	395 (26.82)
**Age (years)**	
*Mean ±* SD	46.99 ± 13.98
Median [IQR]	45 [35–59]
Minimum-Maximum	25–84
**Years of medical practice, median [IQR]**	
*Specialist (n = 1147)*	
<5	135 (11.77)
5–9	164 (14.30)
10–19	299 (26.07)
≥20	549 (47.86)
*Resident (n = 326)*	
First year	55 (16.87)
Second year	76 (23.31)
Third year	88 (26.99)
Fourth year	64 (19.63)
Fifth year	43 (13.19)
**Membership to scientific societies, n (%)**	
SIAARTI	438 (29.74)
SIOT	612 (41.55)
SIR	112 (7.60)
SIMFER	311 (21.11)
**Type of healthcare facility clinicians work for, n (%)**	
Outpatient territorial ambulatory within the local health authority	75 (5.09)
Public hospital	370 (25.12)
Hospital within the local health authority	133 (9.03)
University Hospital	440 (29.87)
Not accredited clinics	130 (8.83)
IRCCS private	51 (3.46)
IRCCS public	67 (4.55)
Accredited private hospital	199 (13.51)
Other	8 (0.54)
**Number of OA patients encountered by the clinicians surveyed monthly, n (%) ^a^**	
0–10	117 (7.99)
11–25	218 (14.89)
26–50	435 (29.71)
51–75	220 (15.03)
76–100	260 (17.76)
>100	214 (14.62)
Mean ± SD	62.91 ± 46.79
Median [IQR]	50 [30–99]
Minimum–maximum	0–200
**Patients with joint OA encountered by the clinicians surveyed monthly, (%)**	
Knee	31.1
Hip	21.7
Lumbar	17.7
Cervical	9.15
Hand/wrist	4.49
Ankle	2.73
Poli-arthritis	10.9
Other	1.87
**Number of knee OA patients encountered by the clinicians surveyed monthly, n (%)**	
0–10	418 (28.38)
11–25	455 (30.89)
26–50	392 (26.61)
51–75	108 (7.33)
76–100	62 (4.21)
>100	38 (2.58)
Mean ± SD	29.34 ± 28.78
Median [IQR]	20 [10–40]
Minimum–Maximum	0–200
**Causes of knee OA in patients encountered by the clinicians surveyed, n (%)**	
Primary degenerative OA	1412 (95.86)
Post-traumatic OA (including surgery)	413 (28.04)
OA secondary to systemic conditions (e.g., arthritis, gout)	185 (12.56)
Other	20 (1.36)
Kellgren-Lawrence grade * in knee OA patients encountered by the clinicians surveyed, (%)	
I	1.9
II	47.7
III	47.7
IV	2.8
**Patients with a prior diagnosis of knee OA encountered by the clinicians surveyed, n (%) ^b^**	
0–10	115 (9.74)
11–25	145 (12.28)
26–50	435 (36.83)
51–75	243 (20.58)
76–100	243 (20.58)
Mean ± SD	50.27 ± 26.97
Median [IQR]	50 [30–70]
Minimum–Maximum	0–100
**Clinicians making knee OA diagnosis in patients encountered by the clinicians surveyed, (%)**	
Orthopedics	47.3
Rheumatologist	7.9
Physiatrist	9.5
Radiologist	10.6
General practitioner	22.4
Pain therapist	2
Other	0.4
**Reason why patients with a diagnosis of knee OA seek consultation with the clinicians surveyed, %**	
Pain	64.1
Functional disability	21.9
Inflammation	11
Other	3.1

^a^ Respondents were 1464; ^b^ Respondents were 1181; * Kellgren–Lawrence grading approach is employed to classify the severity of KOA into five grades (grade 0 denotes healthy joints in which the radiographic features of knee OA do not exist; grade I denotes doubtful KOA, grade II denotes mild KOA, grade III denotes moderate KOA, grade IV denotes severe KOA).

## Data Availability

The database used and analyzed during the current study, as well as raw data from surveys, will be available upon reasonable request to SIAARTI at info@siaarti.it.

## Supplementary Materials

The following supporting information can be downloaded at: https://www.mdpi.com/article/10.3390/healthcare12202077/s1, Clinician Questionnaire.

## Abbreviations

ANMAR: Italian Association of Rheumatological Patients; APMARR, Italian Association of People with Rheumatological and Rare Diseases; COPD, chronic obstructive pulmonary disease; COXIB, cyclo-oxygenase inhibitor; GDPR, General Data Protection Regulation; GP(s), general practitioner(s); IAHA, intra-articular hyaluronic acid; IACS, intra-articular corticosteroids; IQR, interquartile range; IRCCS, Institute for Research and Care in Scientific Areas; ISS, Istituto Superiore di Sanità; KOA, knee osteoarthritis; NSAIDs, non-steroidal anti-inflammatory drugs; OA, osteoarthritis; OARSI, OA Research Society International; PRP, platelet-rich plasma; SD, standard deviation; SIAARTI, Italian Society of Anesthesia, Analgesia, Resuscitation and Intensive Care; SIMFER, Italian Society of Physical and Rehabilitative Medicine; SIOT, Italian Society of Orthopedics and Traumatology; SIR, Italian Society of Rheumatology; SYSADOA, symptomatic slow-acting drugs for osteoarthritis.
