# Supplementary material for: Pain Management in Knee Osteoarthritis: Insights from an Exploratory Online Survey of Italian Patients and Physicians
Source: Healthcare (Basel). 2024 Oct 18;12(20):2077. doi: 10.3390/healthcare12202077 (PMC11507079; doi:10.3390/healthcare12202077)
Supplement: Supplementary file 1 [file healthcare-12-02077-s001.zip › healthcare-3103172-supplementary.pdf]

SUPPLEMENTARY MATERIALS

Clinician Questionnaire

Section 1: Demographic Items

Q1: Please indicate your gender:
Q1a: male
Q1b: female
Q1c: I prefer not to declare.

Q2: Please indicate your age:

Q3: Please indicate if you are:
Q3a: specialist/resident in orthopedics and traumatology
Q3b: specialist/resident in anesthesiology
Q3c: specialist/resident in rheumatology
Q3d: specialist/resident in physical medicine and rehabilitation

Q4: Please indicate how many years of medical practice did you spend as a specialist:
Q3a: <5 years
Q3b: 5–10 years
Q3c: 10–20 years
Q3d: >20 years

Q5: Please indicate your year of residency:
Q5a: first
Q5a: second
Q5a: third
Q5a: fourth
Q5a: fifth

Q6: Please indicate your working geographical area:
Q6a: Northern Italy
Q6b: Central
Q6c: Southern Italy (including islands, namely Sicily and Sardinia)

Q7: Please indicate the healthcare facility you are currently working for:
Q7a: Outpatient territorial ambulatory within the local health authority
Q7b: Public hospital
Q7c: Hospital within the local health authority
Q7d: University hospital
Q7e: Not accredited clinics
Q7f: IRCCS private
Q7g: IRCCS public
Q7h: Accredited private hospital
Q7i: Other

Q8: Please indicate how many patients with osteoarthritis (OA) you see monthly:

Q9: With reference to the OA patients you encounter, please indicate the percentage of patients based on the affected joint:
Q9a: knee
Q9b: hip
Q9c: lumbar
Q9d: cervical
Q9e: hand/wrist
Q9f: ankle
Q9g: poli-arthritis
Q9h: other

Q10: Please indicate how many patients with knee OA you see monthly:

Section 2: Clinical profile of the patients encountered by the participant clinicians in their daily practice.

Q11: Which are the main causes of knee OA in the patients you have encountered?
Q11a: primary degenerative OA
Q11b: post-traumatic (including surgery)
Q11c: secondary to systemic conditions (gout, arthritis)
Q11d: other

Q12: When the patient refers to you for the first time, which Kellgren-Lawrence grade is more frequently observed?
Q12a: I
Q12b: II
Q12c: III
Q12d: IV

Q13: How many patients with knee OA refer to you with a previous diagnosis?

Q14: In the case of patients with a prior diagnosis, who was the physician making the first diagnosis?
Q14a: general practitioner
Q14b: orthopedics
Q14c: rheumatologist
Q14d: physiatrist
Q14e: pain therapist
Q14f: radiologist
Q14g: other

Q15: Why does a patient with a prior diagnosis of knee OA refer to you? Please indicate for each cause a percentage:
Q15a: pain
Q15b: functional disability

Q15c: inflammation
Q15d: other
Q16: Please indicate the proportion of patients suffering from knee OA who present moderate-to-severe pain:

Q17: The moderate-to-severe pain associated with knee OA is present:
Q17a: continuously
Q17b: mostly upon load but not at rest
Q17c: both
Q17d: other

Q18: On average, how much time elapses between the diagnosis of pain associated with knee OA and the start of analgesic therapy?
Q18a: Immediately
Q18b: <1 month
Q18c: 1–3 months
Q18d: 3–6 months
Q18e: 3–12 months
Q18f: >12 months

Q19: Among the patients with previously diagnosed knee OA and moderate-to-severe pain you have encountered monthly, how many have been prescribed non-pharmacological therapy/topical NSAIDs?
Q19a: none
Q19b: <10%
Q19c: 10–25%
Q19d: 25–50%
Q19e: 50–75%
Q19f: >75%

Q20: Among the patients with previously diagnosed knee OA and moderate-to-severe pain you have encountered monthly, how many have been prescribed paracetamol/oral NSAIDs/COXIB?
Q20a: none
Q20b: <10%
Q20c: 10–25%
Q20d: 25–50%
Q20e: 50–75%
Q20f: >75%

Q21: Among the patients with previously diagnosed knee OA and moderate-to-severe pain you have encountered monthly, how many have been prescribed weak/strong opioids?
Q21a: none
Q21b: <10%
Q21c: 10–25%
Q21d: 25–50%
Q21e: 50–75%
Q21f: >75%

Q22: Among the patients with previously diagnosed knee OA and moderate-to-severe pain you have encountered monthly, how many have been prescribed SYSADOA?
Q22a: none
Q22b: <10%
Q22c: 10–25%
Q22d: 25–50%
Q22e: 50–75%
Q22f: >75%

Q23: Among the patients with previously diagnosed knee OA and moderate-to-severe pain you have encountered monthly, how many have been prescribed intra-articular hyaluronic acid?
Q23a: none
Q23b: <10%
Q23c: 10–25%
Q23d: 25–50%
Q23e: 50–75%
Q23f: >75%

Q24: Among the patients with previously diagnosed knee OA and moderate-to-severe pain you have encountered monthly, how many have been prescribed intra-articular corticosteroids?
Q24a: none
Q24b: <10%
Q24c: 10–25%
Q24d: 25–50%
Q24e: 50–75%
Q24f: >75%

Q25: Among the patients with previously diagnosed knee OA and moderate-to-severe pain you have encountered monthly, how many have been prescribed intra-articular platelet-rich plasma/stem cells?
Q25a: none
Q25b: <10%
Q25c: 10–25%
Q25d: 25–50%
Q25e: 50–75%
Q25f: >75%

Section 3: Prescribing habits and degree of satisfaction of clinicians when managing moderate-to-severe pain associated with knee OA

Q26: Which interventions do you more frequently employ to manage moderate-to-severe pain associated with knee OA (please indicate the three most frequent):

Q26a: non-pharmacological therapy

Q26b: topical NSAIDs
Q26c: SYSADOA
Q26d: oral NSAIDs/paracetamol
Q26e: COXIB
Q26f: weak opioids
Q26g: strong opioids
Q26h: intra-articular hyaluronic acid
Q26i: intra-articular corticosteroids
Q26l: intra-articular platelet-rich plasma/stem cells

Q27: Please indicate your degree of satisfaction with the pain relief achieved by topical NSAIDs/paracetamol/SYSADOA in the treatment of patients with moderate-to-severe pain associated with knee OA:

Q27a: very unsatisfied
Q27b: unsatisfied
Q27c: neutral
Q27d: satisfied
Q27e: very satisfied

Q28: Please indicate your degree of satisfaction with the pain relief achieved by oral NSAIDs, COXIB, and weak and strong opioids in the treatment of patients with moderate-to-severe pain associated with knee OA:

Q28a: very unsatisfied
Q28b: unsatisfied
Q28c: neutral
Q28d: satisfied
Q28e: very satisfied

Q29: Please indicate your degree of satisfaction with the pain relief achieved by intra-articular hyaluronic acid, intra-articular corticosteroids, and intra-articular platelet-rich plasma/stem cells in the treatment of patients with moderate-to-severe pain associated with knee OA:

Q29a: very unsatisfied
Q29b: unsatisfied
Q29c: neutral
Q29d: satisfied
Q29e: very satisfied

Q30: How much do patient expectations matter in your therapeutic choice when dealing with the pain associated with knee OA?
Q30a: nothing
Q30b: little
Q30c: enough
Q30d: very much

Patient Questionnaire

Section 1: Demographic Items

Q1: Please indicate your gender:
Q1a: male
Q1b: female
Q1c: I prefer not to declare.

Q2: Please indicate your age:

Q3: Please indicate your living area:
Q3a: Northern Italy
Q3b: Central
Q3c: Southern Italy (including islands, namely Sicily and Sardinia)

Q4: Please indicate your membership:
Q4a: ANMAR
Q4b: APMARR

Section 2: Patient clinical profile

Q5: How long have you been suffering from osteoarthritis (OA)?
Q5a: <6 months
Q5b: 6–12 months
Q5c: 1–3 years
Q5d: 3–5 years
Q5e: >5 years

Q6: Please indicate which joints are affected from OA:
Q6a: knee
Q6b: hip
Q6c: cervical
Q6d: lumbar
Q6e: shoulder
Q6f: elbow
Q6g: ankle
Q6h: hand/wrist

Q7: Please indicate if you are suffering from knee OA at one or both knees:
Q7a: one knee
Q7b: both knees

Q8: Beyond knee OA, are you suffering from other diseases?
Q8a: no
Q8b: hypertension
Q8c: diabetes

Q8d: cardiovascular diseases
Q8e: obesity
Q8f: other (please specify)

Q9: Please rate how much each of the following symptoms impact your daily life:

	Score 0 (no impact)	1	2	3	4	5	6	7	8	9	Score 10 (burdensome)
Pain											
Joint stiffness											
Mobility issues											
Tiredness											
Joint swelling											
Loss of flexibility											
Sleep disorders											
Joint deformity											
Crackling knee (crepitus)											

Q10: Please rate how much pain impacts the following activities:

	Score 0 (no impact)	1	2	3	4	5	6	7	8	9	Score 10 (burdensome)
Physical activity											
Working activity											
Social activities											
Emotion/psychological dimension											

Q11: Please indicate the intensity of your pain on a scale of 0–10:

Section 3: Patient journey: from diagnosis to therapy

Q12: Please indicate your age at first diagnosis of knee OA:

Q13: Please indicate which specialist made the first knee OA diagnosis:
Q13a: general practitioner
Q13b: orthopedics
Q13c: rheumatologist
Q13d: physiatrist
Q13e: pain therapist
Q13f: radiologist
Q13g: other (please specify)

Q14: Please indicate how much time elapses between the first visit to the confirmed diagnosis:
Q14a: immediately
Q14b: <1 month
Q14c: 1–3 months
Q14d: 3–6 months
Q14e: >6 months

Q15: Please indicate by which means knee OA diagnosis was made:
Q15a: X-rays
Q15b: magnetic resonance
Q15c: echography
Q15d: physician objective examination
Q15e: I do not remember

Q16: When you received your first diagnosis of knee OA, did you receive all the necessary information regarding the disease, lifestyle, therapies, and so on?
Q16a: Yes
Q16b: No
Q16c: I do not remember

Q17: Please indicate the number of physicians you have encountered before receiving the knee OA diagnosis:

Q18: On average, how much time elapses between diagnosis of pain associated with knee OA and your first prescription of analgesic medications?
Q18a: Immediately
Q18b: <1 month
Q18c: 1–3 months
Q18d: 3–6 months
Q18e: 3–12 months
Q18f: >12 months

Q19: During your treatment journey, how much did you feel listened to and involved in the choice of therapy to manage pain associated with knee OA?
Q19a: not at all
Q19b: little
Q19c: enough
Q19d: very much

Q20: Please indicate the most relevant gaps you have dealt with during your patient journey:

<u>Section 4: Prescribed analgesic medications and patient degree of satisfaction</u>

Q21: Please indicate which of the following treatments you are currently taking to manage the pain associated with knee OA:
Q21a: non-pharmacological therapy
Q21b: topical NSAIDs
Q21c: paracetamol
Q21d: oral NSAIDs
Q21e: opioids
Q21f: intra-articular hyaluronan
Q21g: intra-articular corticosteroids
Q21h: currently, I am not treating the pain
Q21i: other (please specify)

Q22: Please indicate the degree of satisfaction with the prescribed analgesic therapy:
Q22a: not at all
Q22b: little
Q22c: enough
Q22d: very much
Q22e: currently, I am not treating the pain

Q23: If you consider yourself unsatisfied, please indicate the reason:
